# Invasive carcinoma of urinary bladder in a patient with a spinal cord injury with non-functioning Brindley sacral anterior root stimulator: a case report

**DOI:** 10.1186/1757-1626-1-137

**Published:** 2008-09-01

**Authors:** Subramanian Vaidyanathan, Bakul M Soni, Paul Mansour, Gurpreet Singh, Peter L Hughes

**Affiliations:** 1 Regional Spinal Injuries Centre, District General Hospital, Southport, PR8 6PN, UK; 2 Department of Cellular Pathology, District General Hospital, Southport PR8 6PN, UK; 3 Department of Urology, District General Hospital, Southport PR8 6PN, UK; 4 Department of Radiology, District General Hospital, Southport, PR8 6PN, UK

## Abstract

**Background:**

Anterior sacral root stimulation combined with sacral posterior rhizotomy restores bladder function in spinal cord-injured patients suffering from hyperactive bladder. After successful implantation of bladder stimulator, urinary infection rate decreases, and patients are able to get rid of indwelling urinary catheters, which in turn reduce the risks for vesical malignancy. We present a spinal cord injury patient with non-functioning Brindley sacral anterior root stimulator, who developed carcinoma of urinary bladder.

**Case presentation:**

A Caucasian male, who was born in 1943, sustained paraplegia at T-4 (ASIA-B) in 1981. This patient underwent implantation of sacral anterior root stimulator in September 1985. The bladder stimulator started giving trouble since 1996 and the patient went back to using indwelling urethral catheter. In August 2006, this patient passed blood in urine after a routine change of indwelling catheter. Cystoscopy showed unhealthy bladder mucosa. Bladder biopsy revealed carcinoma, which was infiltrating bundles of muscularis propria. Many of the nests showed evidence of squamous differentiation, while others could be transitional or squamous. This patient underwent cystectomy with lymphadenectomy in March 2007 in a hospital nearer his home. Histology showed three nodes involved. This patient has been doing well since the operation.

**Conclusion:**

Occurrence of vesical malignancy in this patient with non-functioning bladder stimulator is a timely reminder to all health professionals, and health care managers that concerted efforts should be made to rectify a non-functioning sacral anterior root stimulator as soon as possible. Otherwise, facilities should be made available in the community for the spinal cord injury patient to use intermittent catheterisation and thereby, avoid permanent indwelling catheter, vesical calculi and urine infections, which are risk factors for bladder cancer.

## Background

Spinal cord injury above the level of sacral micturition centre may induce detrusor hyperreflexia, spasticity of urethral sphincter and consequently, detrusor-sphincter dyssynergia. Anterior sacral root stimulation combined with sacral posterior rhizotomy is a valuable method to restore bladder function in spinal cord-injured patients suffering from hyperactive bladder. After successful implantation of anterior sacral root stimulator, bladder capacity and compliance increase dramatically. As a consequence, urinary infection rate decreases [1-3]. Further, these patients in whom the implant is working satisfactorily, are able to get rid of indwelling urinary catheters as many of them are continent and those, who are not continent, wear a penile sheath.

Spinal cord injury patients with neuropathic bladder are at an increased risk of developing bladder malignancies [4,5]. Risk factors for bladder cancer in spinal cord injury patients remain controversial, but the duration of neurogenic bladder and the voiding mode appear to be the main risk factors [6]. Indwelling catheter is a significant independent risk factor for the increased risk of and mortality caused by bladder cancer in the spinal cord injury population [7]. Following successful implantation of a sacral anterior root stimulator in a spinal cord injury patient, it is likely that the decreased incidence of urinary infection and absence of long term indwelling catheters would reduce the chances of neoplastic changes in the neuropathic bladder. This assumption is supported by the fact that a search in PubMed showed no report of bladder cancer in a spinal cord injury patient, who had undergone implantation of sacral anterior root stimulator.

We present a spinal cord injury patient with non-functioning Brindley sacral anterior root stimulator, who developed carcinoma of urinary bladder. This patient with non-functioning sacral anterior root stimulator started retaining urine in the bladder and subsequently, required permanent indwelling catheter, as facilities for intermittent catheterisation were not available in the community. Occurrence of invasive carcinoma of urinary bladder showing squamous differentiation in this patient raises the question as to whether a non-functioning bladder stimulator exposes the spinal cord injury patient to the risk of malignancy if the patient does not empty the bladder satisfactorily and long-term indwelling catheter drainage is required.

## Case presentation

A Caucasian male, who was born in 1943, sustained paraplegia at T-4 (ASIA-B) in a road traffic accident in 1981. This patient underwent implantation of sacral anterior root stimulator in September 1985. Although he made an uneventful recovery initially after the operation, this patient developed constant headaches and a fluctuant mass appeared under the scar. Re-exploration of lumbar laminectomy wound was performed in September 1985. There was a continuing leak of cerebrospinal fluid along the electrodes. Surgical repair was carried out to seal the cerebrospinal fluid leak from the meninges. Following the second operation, the patient's recovery was entirely uneventful and at the time of discharge, the stimulator was working well producing a good flow of urine when switched on.

In March 1987, surgery, consisting of two separate procedures, was carried out under the same anaesthetic. Firstly, a complete anatomical transaction of the spinal cord was performed at a spinal level of T-6 in an attempt to remove all propagation of noxious inputs from below T-6. Secondly, spasms in the lower abdomen appeared to have been due to failure to divide sufficient dorsal rami at the time of insertion of the stimulator, so the S-2 and S-3 dorsal roots were divided at their entry into the conus medullaris. Postoperatively, the pain was subjectively altered into a paraesthesia type of sensation, but there was still pain associated with the filling of the lower bowel, this almost certainly representing sympathetic nervous system transmission of pain outside the spinal cord. The lower abdominal and leg spasms were abolished. The function of the stimulator itself was much improved with a good stream and complete emptying of the bladder as well as improvement in bowel function, although the patient did have a period of ileus post-operatively.

In July 1989, exploration of the bladder stimulator receiver was carried out as the system had been mal-functioning for two weeks. One of the wires had become disconnected, and this fault was rectified. Initially, it was difficult to activate the bladder stimulator with the external transmitter; therefore, the patient was re-catheterised. However, accurate localisation of the subcutaneously positioned receiver enabled full function of bladder stimulator.

The bladder stimulator started giving trouble since 1996; tuning became very difficult. When the patient used bladder stimulator, even on programme 2 to empty his bladder, this caused a bowel evacuation. Although programme 2 was successful in emptying his bladder he had gone back to an indwelling catheter. This patient was not using the programmable box at all because of the problems with faecal incontinence. When he went out socially, stimulating the bladder to empty via a sheath into a leg bag caused bowel evacuation.

**Figure 1:**
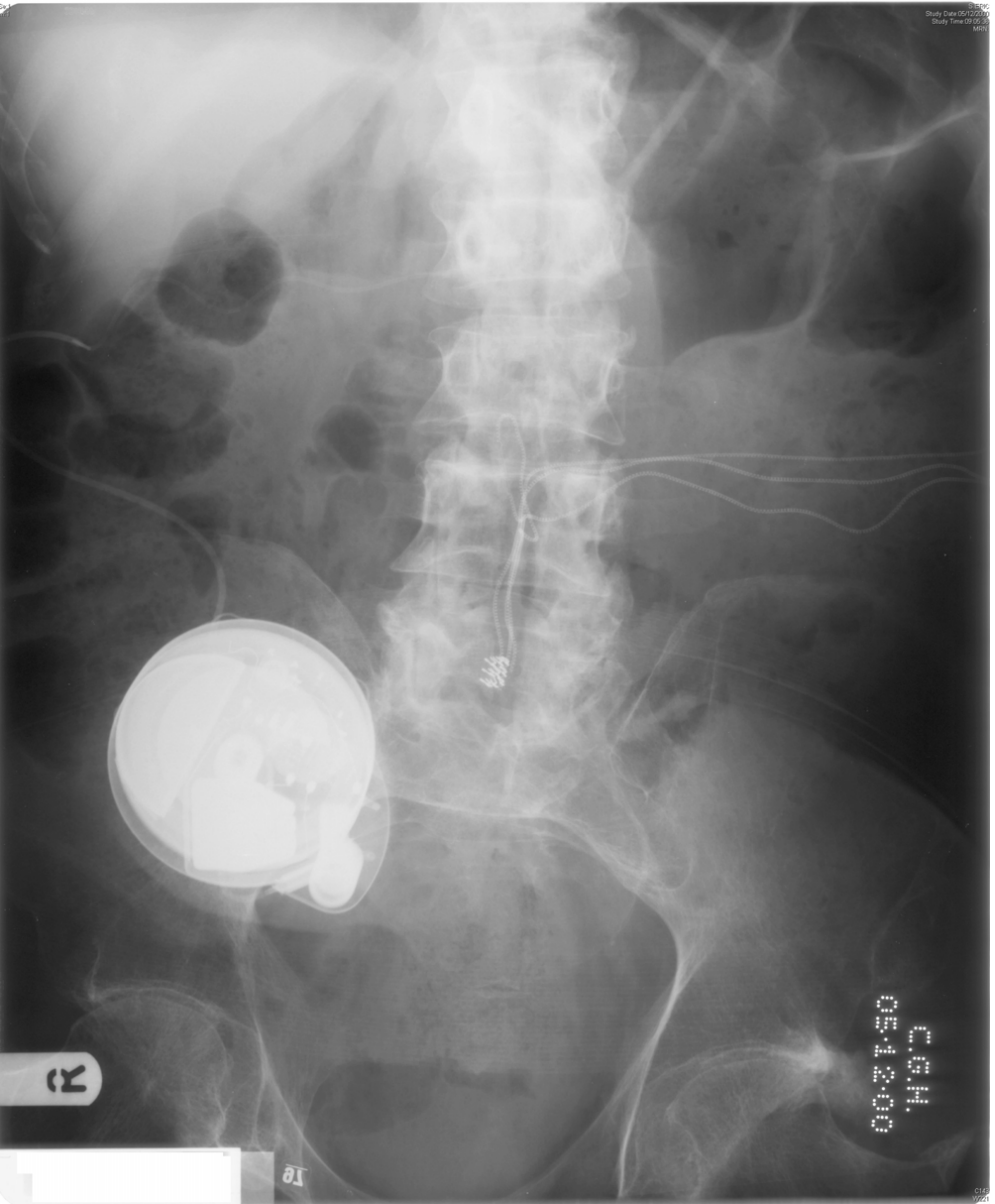
X-ray of abdomen, taken on 05 December 2000, shows baclofen pump on the right side. The electrodes of sacral anterior root stimulator can be seen in midline in the lumbar area. The wires for the sacral anterior root stimulator travel to the left side of abdomen where the receiver is located.

**Figure 2:**
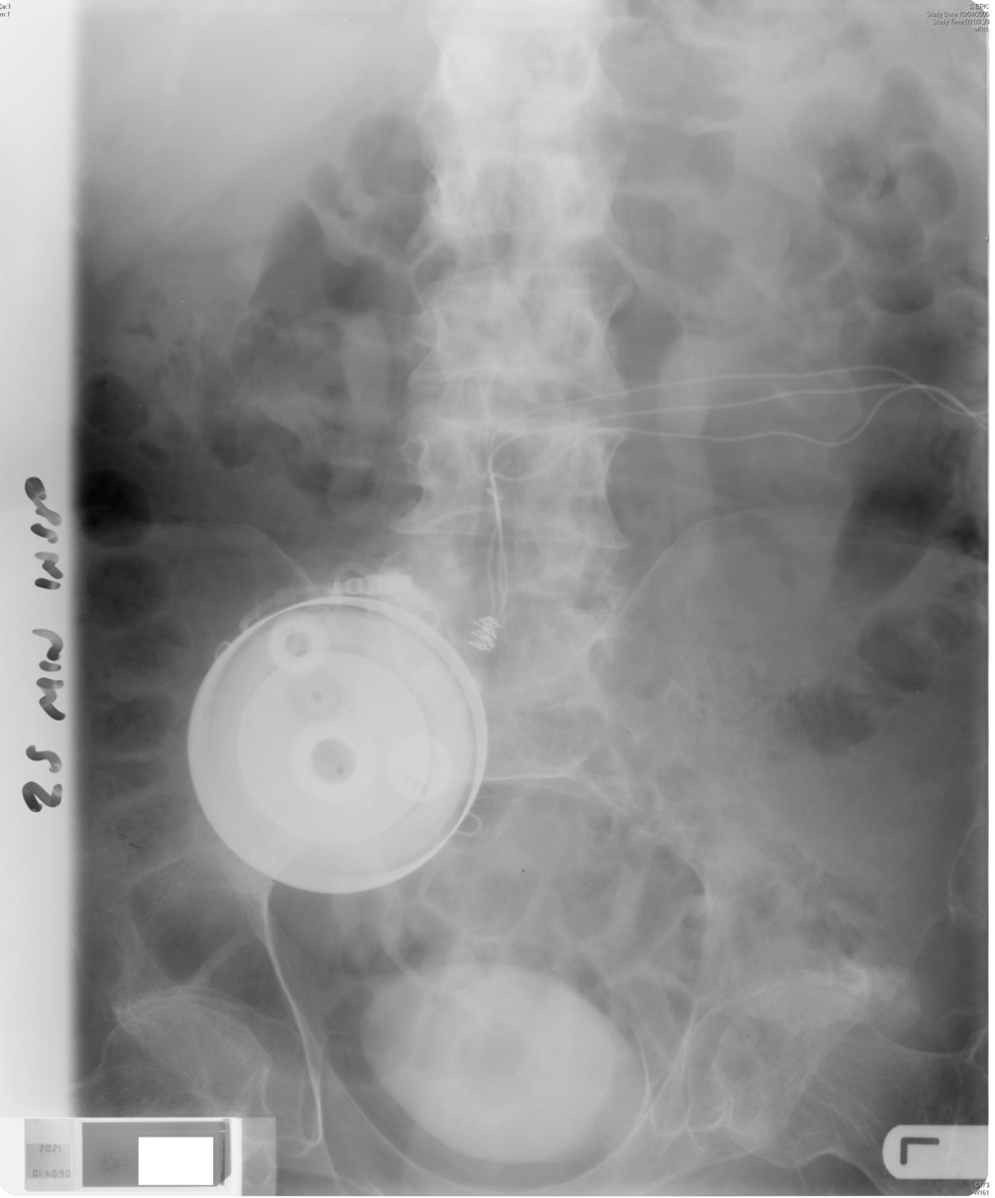
Intravenous urography, performed on 10 April 2006: Twenty minutes film showed smooth bladder outline. The electrodes and wires of sacral anterior root stimulator were seen.

X-ray of abdomen taken in December 2000 showed no opaque calculi in kidneys and bladder (Figure 1). Colostomy was performed in December 2000. Intravenous urography, carried out in March 2002, showed normal kidneys, pelvicalyceal system and ureters. The bladder outline appeared normal with catheter in situ. Intravenous urography, performed in April 2006, showed normal kidneys, ureters and bladder (Figure 2).

**Figure 3:**
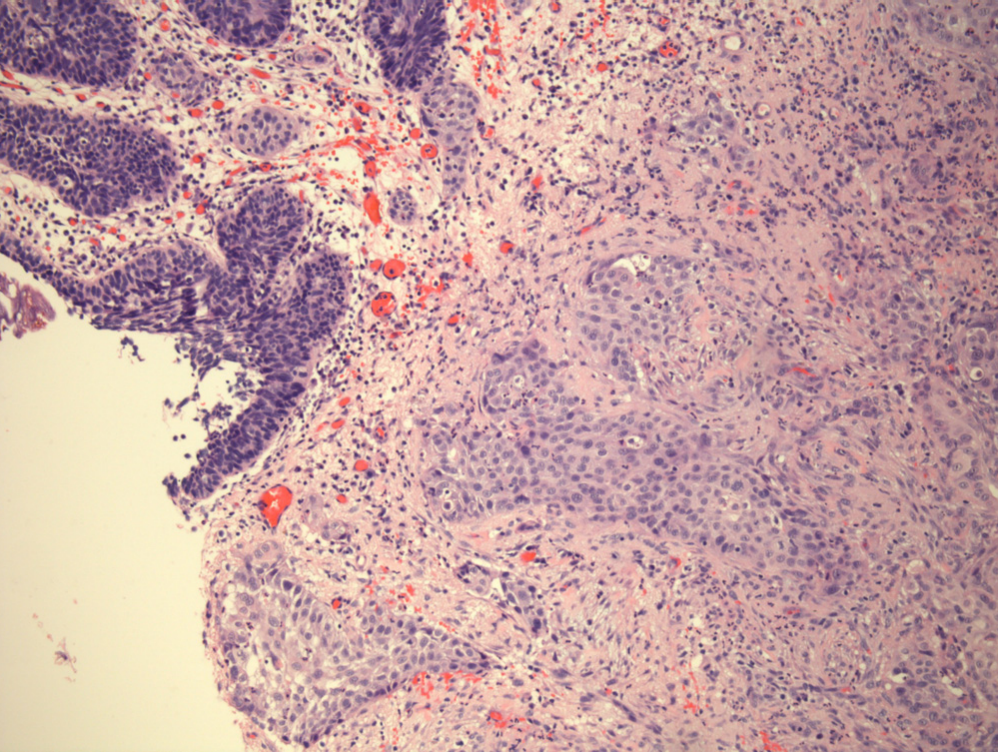
Photomicrograph of bladder biopsy shows invasive carcinoma (right and bottom) with evidence of squamous differentiation, and overlying carcinoma-in-situ (top and left).

In July 2006, the urethral catheter kept blocking; three catheters were fitted in eleven days. Cystoscopy revealed several calculi in the bladder; bladder mucosa was congested. Electrohydraulic lithotripsy was performed. In August 2006, this patient passed blood in urine after a routine change of indwelling catheter. Cystoscopy showed unhealthy bladder mucosa. Bladder biopsy revealed carcinoma, which was infiltrating bundles of muscularis propria. Many of the nests showed evidence of squamous differentiation, while others could be transitional or squamous. It was possible that the tumour was a squamous carcinoma rather than a transitional cell carcinoma showing squamous differentiation. One of the other biopsies showed squamous metaplasia of the surface epithelium in which there was moderate dysplasia, probably falling short of carcinoma in situ (Figure 3). Computerised tomography of abdomen and pelvis revealed normal appearance of the liver, kidneys and pancreas. Urinary bladder was empty with catheter in situ. There was no evidence of any infiltration into the perivesical fat. This patient underwent cystectomy with lymphadenectomy in March 2007 in a hospital nearer his home. Histology showed three nodes involved. The tumour was not through the bladder wall; it was Grade 3. This patient has been doing well since the operation; he went on holidays to Wales with his wife in a caravan during summer of 2008.

## Discussion

It is possible that use of sacral anterior root stimulator for emptying of neuropathic bladder may confer protection against development of vesical malignancy. The likely factors which decrease the risk of vesical malignancy in patients with a satisfactorily functioning stimulator are: (1) decreased incidence of urine infection, and (2) absence of permanent indwelling catheter following a successful implantation of sacral root stimulator. Further, electrical stimulation of urinary bladder *per se *may have an effect on cell proliferation [8,9]. Low-intensity, intermediate-frequency (100–300 kHz), alternating electric fields, delivered by means of insulated electrodes, were found to have a profound inhibitory effect on the growth rate of a variety of human and rodent tumor cell lines and malignant tumors in animals. This effect, shown to be nonthermal, selectively affects dividing cells while quiescent cells are left intact. The electrical fields arrest cell proliferation and destroy cells undergoing division [9].

This patient with a non-functioning Brindley bladder stimulator had been using long-term catheter and developed vesical calculi. Ten years after the stimulator started malfunctioning, invasive carcinoma showing squamous differentiation was detected in the neuropathic bladder. Indwelling catheters and chronic urine infections were common in spinal cord injury patients, who developed bladder cancer, which was urothelial cancer in 81% and squamous cell cancer in 19% [4]. This case is a timely reminder to all health professionals, and health care managers that concerted efforts should be made to rectify a non-functioning sacral anterior root stimulator as soon as possible. Otherwise, facilities should be made available in the community for the spinal cord injury patient to use intermittent catheterisation and thereby, avoid permanent indwelling catheter, vesical calculi and recurrent urine infections [10].

## Conclusion

Occurrence of invasive carcinoma showing squamous differentiation in urinary bladder of this patient, who had non-functioning sacral anterior root stimulator, is a timely reminder to all health professionals, and health care managers that concerted efforts should be made to rectify a non-functioning bladder stimulator as soon as possible. Otherwise, facilities should be made available in the community for the spinal cord injury patient to use intermittent catheterisation and thereby, avoid permanent indwelling catheter, vesical calculi and urine infections, which are risk factors for cancer in neuropathic bladder.

## Competing interests

The authors declare that they have no competing interests.

## Authors' contributions

SV developed the concept and wrote the draft. GS performed bladder biopsy. PM interpreted bladder biopsy slides. PH reported CT of abdomen. All authors contributed to the manuscript.

## Consent

The leading author discussed publication of the case with the patient and his wife. The patient was happy for us to present his case in the Cases Journal. He gave written consent for publication of his case in the Cases Journal.
